# Reversing the immune ageing clock: lifestyle modifications and pharmacological interventions

**DOI:** 10.1007/s10522-018-9771-7

**Published:** 2018-09-29

**Authors:** Niharika A. Duggal

**Affiliations:** MRC-Arthritis Research UK Centre for Musculoskeletal Ageing Research, Institute of Inflammation and Ageing, Birmingham University, Birmingham, UK

**Keywords:** Ageing, Immunesenescence, Inflammaging

## Abstract

It is widely accepted that ageing is accompanied by remodelling of the immune system, including reduced numbers of naïve T cells, increased senescent or exhausted T cells, compromise to monocyte, neutrophil and natural killer cell function and an increase in systemic inflammation. In combination these changes result in increased risk of infection, reduced immune memory, reduced immune tolerance and immune surveillance, with significant impacts upon health in old age. More recently it has become clear that the rate of decline in the immune system is malleable and can be influenced by environmental factors such as physical activity as well as pharmacological interventions. This review discusses briefly our current understanding of immunesenescence and then focuses on lifestyle interventions and therapeutic strategies that have been shown to restore immune functioning in aged individuals.

## Immune ageing and health

Over the past 250 years life expectancy has increased dramatically and is still increasing at 2 years per decade in most countries. Advancing age is accompanied by a compromised ability of older adults to combat bacterial and viral infections (Gavazzi and Krause 2002; Molony et al. 2017a, b), increased risk of autoimmunity (Goronzy and Weyand 2012), poor vaccination responses (Del Giudice et al. 2017; Lord 2013) and the re-emergence of latent infections to produce conditions such as shingles (Schmader 2001). All of this contributing towards increased morbidity and mortality in older adults (Pera et al. 2015) and indicative of reduced immunity.

Another universal feature of physiological ageing is an increase in circulating levels of pro-inflammatory cytokines (IL-1β, IL-6, IL8, TNFα, IFNγ, and CRP) termed “Inflammaging” (Franceschi et al. 2007). Importantly, a strong association has been reported between elevated pro-inflammatory cytokine levels in older adults and mortality (Cohen et al. 2003), frailty (Cesari et al. 2004), age-related chronic diseases (Ershler and Keller 2000) and cognitive impairment (Yaffe et al. 2003). Inflammaging is multifactorial with some of the factors proposed to contribute to inflammaging including: lifetime antigenic load resulting in oxidative damage; increased DNA damage, accumulation of senescent cells, increased visceral adipose tissue; decline in sex hormones and reduced immune regulation (Baylis et al. 2013; Singh and Newman 2011).

**Fig. 1:**
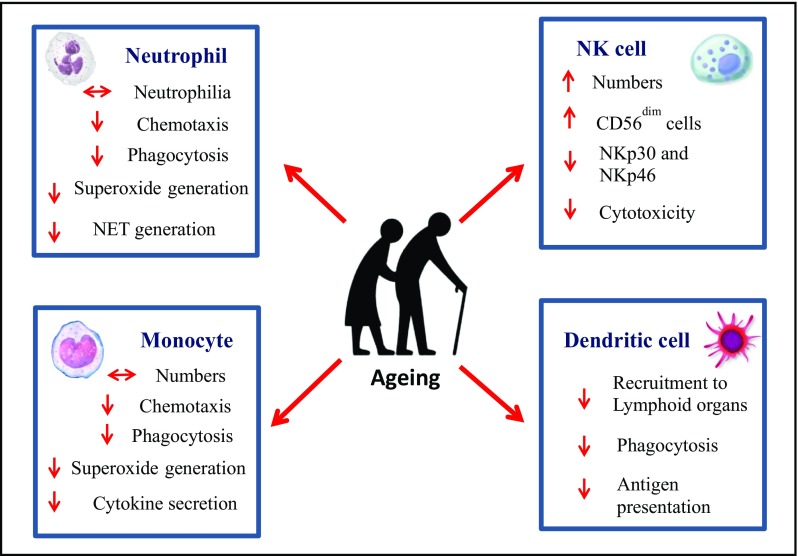
Age related alterations in innate immune cells

Altered immunity with age is the result of remodelling of both the innate and adaptive arms of the immune system, collectively termed immunesenescence. Delaying or reversing the effects of ageing on the immune system could be extremely beneficial in maximising health and improving quality of life in older adults (Dorshkind et al. 2009). For the cells of the innate immune response the literature is clear that their numbers increase with age but their function upon challenge with pathogens declines. Thus age-associated defects have been observed in neutrophils, monocytes/macrophages, NK cells and dendritic cells (Fig. 1). Neutrophils are the primary immune defence against bacterial and fungal infections and recent studies have revealed that in addition to the well documented reduction in phagocytosis and ROS generation, these cells also show reduced chemotaxis to a range of stimuli (Sapey et al. 2014) and a reduced ability to extrude their DNA as NETs to entrap bacteria extracellularly (Hazeldine et al. 2014). Additionally, an age-associated reduction in TLR1 expression on neutrophils has been associated with reduced chemokine (IL8) production, reduced rescue from apoptosis and lower expression of activation markers, resulting from reduced bioenergetics in neutrophils (Qian et al. 2014).

Circulating monocytes play a critical role in defence against invading pathogens being early responders to infection and able to act as antigen presenting cells. Ageing affects the distribution of monocyte subsets with a decline in classical monocytes (CD14^+ve^ CD16^−ve^) and increase in intermediate (CD14^+ve^ CD16^+ve^) and non-classical monocytes (CD14^+ve^ CD16^++ve^) occurs with age (Seidler et al. 2010). Interestingly, non-classical monocytes express high levels of miR-146a and exhibit a senescence associated secretory phenotype (SASP), contributing towards inflammaging (Ong et al. 2018). Also, age-associated changes in platelets also contribute towards inflammatory cytokine production by monocytes (Campbell et al. 2018). However, monocyte cytokine secretion in response to stimulation via TLRs is greatly reduced with age (Metcalf et al. 2017). A notable exception is TLR5 expression that has been reported to increase with age, offering an opportunity to develop improved vaccines for older adults (Qian et al. 2012). Retinoic acid inducible gene 1 (RIG-1) like receptors play a key role in recognition of viral nucleic acids and older human monocytes have intact RIG1 signalling to activate pro-inflammatory cytokines but have a diminished IFN response (Pillai et al. 2016; Molony et al. 2017a). Cytosolic dsDNA receptor AIM2 triggers the inflammasome and is important for control of virus infections (Guo et al. 2015). Recently, defects in AIM2 expression have been reported in monocytes of older adults, impairments in caspase 1 activation upon stimulation of AIM2 has been associated with reduced IL1β secretion (Wang et al. 2016). These alterations in innate immune responses to pathogens with ageing have consequences for the ability of older adults to respond to an infectious challenge. Other monocyte functions such as wound repair (Sebastián et al. 2005) and clearance of apoptotic cells (Aprahamian et al. 2008) are also compromised in older adults.

Dendritic cells play a key role in initiating pathogen-specific adaptive immune responses and have been divided into two subsets—myeloid DCs and plasmacytoid DCs. A reduction in plasmacytoid DCs and unaltered frequency of myeloid DCs have been reported in older adults (Garbe et al. 2012). Recent data in this field has led to further division of myeloid DCs into two subsets; CD1c^+ve^ and CD141^+ve^ mDCs. Ageing is accompanied by a decline in peripheral CD141^+ve^ DCs, whereas the CD1c^+ve^ mDCs remain unaltered (Agrawal et al. 2016). Age associated impairments occur in DC recruitment to lymphoid organs post antigen exposure which has been associated with mitochondrial dysfunction (Chougnet et al. 2015). Surprisingly, the age associated ability of DCs to phagocytose *C.albicans* remains intact (Do Nascimento et al. 2015). Also, the ability of DCs to induce T cell proliferation and IFNγ secretion is impaired in older adults, resulting in impairments in vaccine responses (Panda et al. 2010; Sridharan et al. 2011). Another age associated dysfunction of DCs is in their ability to efficiently activate NK cells which is likely to contribute to impaired tumour immunity (Guo et al. 2014).

NK cells are innate cytotoxic lymphocytes that play an essential role in defence against viral infections and malignancies and they also kill senescent cells thus contributing to delaying the ageing phenotype (Sagiv et al. 2013). Ageing is accompanied by an increase in NK cell numbers, due to expansion of CD56^dim^ NK cells (Le Garff-Tavernier et al. 2010; Almeida-Oliveira et al. 2011). CMV seropositivity and proinflammatory status (Campos et al. 2014) are contributors towards altered NK cell subset distribution. The expression of NK cell receptors; NKp46 ^and^ NKp30 has been shown to decline with age, whereas NKG2D expression remains unaltered (Solana et al. 2014), as NKG2D is required for the killing of senescent cells (Sagiv et al. 2016) this may affect the killing ability of NK cells towards senescent cells, though this has not yet been shown. In this context, NK cell cytotoxicity towards cancer cells is mediated by granule exocytosis and is reduced with age (Almeida-Oliveira et al. 2011; Hazeldine et al. 2012), due to reduced release of perforin (Hazeldine et al. 2012). In contrast, NK cell mediated antibody dependent cell cytotoxicity (ADCC) is preserved with age (Lutz et al. 2005).

**Fig. 2:**
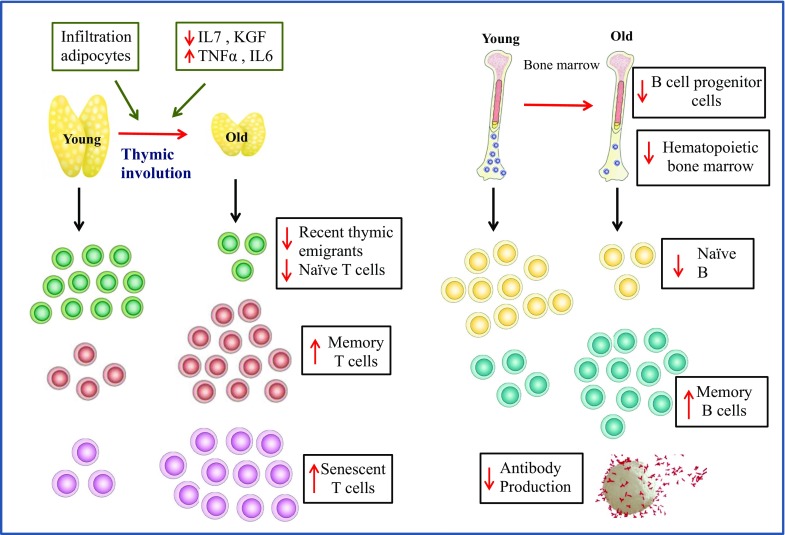
Age related alterations in adaptive immune cells

Myeloid derived suppressor cells (MDSCs) are known to play an important role in suppression of T cell responses (Gabrilovich and Nagaraj 2009). Importantly, ageing is accompanied by an increase in MDSCs, which has been linked with a higher incidence of cancer and chronic inflammation in aged individuals (Enioutina et al. 2011). In the adaptive immune system, the effects of age are also significant (Fig. 2). The thymus is devoted to T lymphocyte differentiation and maturation and ageing is associated with atrophy of the thymus (Mitchell et al. 2010). In humans, thymic atrophy involves a decrease in both stromal and thymocyte cellularity with infiltration of adipocytes, loss of tissue organisation, reduced levels of cytokines and hormones essential for thymopoiesis (e.g. IL-7, KGF and Ghrelin) and upregulation of thymosuppressive cytokines (e.g. IL-6, TNFα) with age (Palmer 2013; Ventevogel and Sempowski 2013). The net outcome of thymic involution is reduced naïve T cell output (Haines et al. 2009) which compromises the ability to respond to new pathogens and vaccines. Other hallmarks of T cell immunesenescence include: accumulation of CD28^−ve^ CD57^+ve^ T cells with shortened telomeres and reduced proliferative capacity (Strioga et al. 2011), which also acquire NK cell receptors such as KLRG1 (Weng et al. 2009) increasing risk of autoimmune responses; skewing of T cell responses towards Th17 cell differentiation (Ouyang et al. 2011). Regulatory CD4^+ve^CD25^+ve^Foxp3^+ve^ T cells play a pivotal role in maintaining immune homeostasis by suppressing immune responses. Ageing is associated with an increase in the frequency of T_regs_, which correlates with increasing incidence of cancer in older adults (Hou et al. 2017).

As with T cells there is a decline in the frequency of naïve B cells (CD27^−ve^ IgD^+ve^) and an increase in memory B cells has also been reported (Colonna-Romano et al. 2006) (Fig. 2). Ageing is accompanied by poor vaccination responses, likely due to reduced B cell and T cell activity (Siegrist and Aspinall 2009). Older adults generate tenfold fewer antibody secreting cells relative to young individuals on antigenic stimulation (Kogut et al. 2012). Additionally, the antibodies produced by aged B cells have lower affinity and fewer antibodies are pathogen specific (Howard et al. 2006). A decline in CD4 T cell and dendritic cell functioning, along with intrinsic changes in B cells resulting in age-associated reduction in number and size of germinal centres are all contributing factors towards the decline in antibody production by older individuals with age (Frasca and Blomberg 2009). Further, an age associated numerical and functional deficit in a novel subset of immunosuppressive CD19^+ve^ CD24^hi^ CD38^hi^ B cells has been recently reported, which might be a factor contributing towards increased risk of systemic autoimmunity with advancing age (Duggal et al. 2012).

As the immune system does not operate in isolation and can be modified by a broad range of environmental signals, we now consider how modification of lifestyle could be used to improve on the reduced immune responses of older adults.

## Impact of lifestyle factors on immunesenescence and inflammaging

### Physical activity

Regular physical activity has been associated with major health benefits including; reduced risk of cardiovascular diseases, diabetes, stroke, sarcopenia and mortality (Shepherd et al. 2015). However, ageing is accompanied by a sharp decline in both duration and intensity of physical activity and the majority of older adults fail to meet the World Health organisation (WHO) recommended guidelines of 150 min of aerobic exercise per week (World Health Organisation 2010).

Exercise in older adults has been associated with lower levels of pro-inflammatory cytokines such as IL6, TNFα (Gleeson et al. 2011). Physical activity exerts an anti-inflammaging effect via several mechanisms. Fat mass increases with age, which has been associated with low-grade chronic inflammation. This is due in large part to the pro-inflammatory cytokines secreted by fat, termed adipokines. Levels of pro-inflammatory adipokines such as leptin and visfatin increase with age whereas anti-inflammatory adiponectin decreases (Gulcelik et al. 2013). In addition, adipose tissue contains immune cells including macrophages which themselves secrete pro-inflammatory cytokines (Vieira-Potter 2014). Regular physical activity has been associated with a reduction of visceral and abdominal fat (Ross and Bradshaw 2009) resulting in a reduction of pro-inflammatory cytokine levels. Skeletal muscle itself is also a major source of cytokines, termed myokines in the physiology literature (Hoffmann and Weigert 2017). Exercising muscle transiently secretes less IL-6 and more anti-inflammatory IL-10 (Steensberg et al. 2003), providing a counter to inflammaging.

Physical activity also has direct effects on immune function, including in older adults. In aged humans intervention studies ranging from 6 weeks up to 10 months, performed 1–6 times per week have reported multiple effects on the immune system including: improved neutrophil chemotaxis (Bartlett et al. 2016) and phagocytosis (Sasaki et al. 2013; lower frequency of CD16^+ve^ monocytes that may be critical in reducing inflammaging (Timmerman et al. 2009); enhanced NK cell cytotoxicity (Bigley et al. 2015), mobilisation of dendritic cells (Suchánek et al. 2010), reduced memory T cells and increased T cell proliferation (Shinkai et al. 1995), increased T cell telomere length (Silva et al. 2016), improved T helper cell functioning (Shimizu et al. 2008) and immature B cell mobilisation (Turner et al. 2016). In a recent study we reported that high levels of physical activity in adulthood has a beneficial effect on thymic output likely to be a result of improved thymic microenvironment (raised levels of IL7 and lower levels of IL6). Additionally, we found the maintenance of peripheral naïve T cell frequency in the active older adults which was associated with higher serum IL15 (Duggal et al. 2018). Others have shown that individuals who perform regular exercise appear to be at a reduced risk of mortality from infections (Lowder et al. 2005) and participation in aerobic exercise for 3 months before the influenza vaccination improved vaccination responses in older adults (Kohut et al. 2004). However it is likely that physical activity needs to be maintained to achieve effects on vaccination responses as a study involving a 45 min brisk walk before vaccination showed no effect (Long et al. 2012). In summary, regular participation in physical activity is a non-invasive, mostly cost neutral anti-immunesenescence and anti-inflammaging therapy.

### Caloric restriction

Restriction of food intake to approximately 30% of ad libitum calorie intake (caloric restriction), without malnutrition, is a robust means of increasing longevity and delaying onset of age-associated diseases in a variety of species including primates (Fontana and Partridge 2015; Mattison et al. 2017). Several of these studies have looked at the impact of CR on immunesenescence and have shown benefits including: maintenance of the thymic microenvironment, higher concentrations of circulating naïve T cells, amelioration of age associated T cell proliferative defects and improved TCR diversity in mice (Yang et al. 2009) and monkeys (Messaoudi et al. 2006). Additionally, age associated accumulation of senescent T cells was not seen in calorically restricted mice (Spaulding et al. 1997). CR can also modulate the cytokine secretion profile of T cells, inducing a decrease in TNFα and IFNγ production by T cells in mice (Nikolich-Zugich and Messaoudi 2005) and suppression of B cell lymphopoiesis in mice (Tang et al. 2016). However, studies investigating the impact of CR on infectious disease susceptibility have yielded contradictory findings. Effros et al. (1991) reported that aged CR mice could mount a vigorous response against influenza, whereas Ritz et al. showed increased susceptibility to infections in aged CR mice (2006).

CR also affects innate immune cells, but these effects have been less well studied and the few studies to date suggest the effects are not universally beneficial. Neutrophil function has been reported to be unaffected by CR in dogs (Greeley et al. 2006), whilst a decline in circulating NK cell frequency (Clinthorne et al. 2013) and NK cell cytotoxicity was seen in CR mice (Ritz et al. 2008). Leptin, an adipocyte secreted hormone plays an important role in maintaining NK cell numbers (Tian et al. 2002). Leptin levels are reduced in CR mice, making it a potential candidate through which immunomodulatory effects of CR are mediated (Clinthorne et al. 2013). Similar to NK cells, reductions in circulating DC numbers and DC progenitors in bone marrow have been reported in CR mice (Duriancik and Gardner 2016).

The high prevalence of centenarians and lower morbidity from age-associated diseases in Okinawans is intriguing evidence supporting the view that CR could also have lifespan and health span benefits in humans (Willcox et al. 2017). The Okinawans have a calorie intake that is much lower than their mainland Japanese peers, related to their beliefs that you should always leave some food on your plate at the end of a meal. Children intake approximately 60% of the recommended calories for a child in the UK and for adults this is 80% (Willcox et al. 2007). Importantly Okinawans have significantly reduced incidence of most cancers that are common in the developed world including breast, prostate and colon cancer and have lower levels of dementia (Willcox et al. 2007). There are currently no data concerning immune function in Okinawans. There are still very few rigorous clinical trials involving CR. A National Institute of Aging sponsored randomized trial of a 2 year caloric restriction regimen in healthy humans (CALERIE) revealed slowing of biological ageing as evidenced by reduced cardiovascular disease risk biomarkers and lower levels of pro-inflammatory cytokines, which has been associated with decline in body fat mass (Das et al. 2017; Ravussin et al. 2015).

Chronic nutrient excess, particularly a high fat diet leads to an increase in systemic inflammation, which over time activates immune cells promoting local chronic inflammation in metabolic cells such as adipocytes and hepatocytes (Franceschi et al. 2017). Thus, it is not surprising that CR, which can result in a reduction in visceral body fat, may be a promising anti-inflammaging intervention. CR has been shown to reduce IL6 levels in old rhesus monkeys (Willette et al. 2010). IL6 is a known thymosuppressive cytokine and reducing its levels by CR could be a contributing factor towards improved thymic output observed in CR animals (Yang et al. 2009). Thus to date, CR has shown impact as a dietary intervention for immunesenescence in animal models but further studies are required to determine its effects on human immune ageing and especially if it is beneficial for all aspects of the immune response.

### Nutrition

Optimal nutrition is an important determinant of healthy ageing and plays a significant role in maintaining immune function. Malnutrition risk significantly increases with age (Elia et al. 2008), possibly contributing to compromised immune function and increased infection susceptibility in older adults. Observational studies have examined the association between reduced expression of pro-inflammatory markers and the Mediterranean diet (low in saturated and high in monounsaturated fats mainly from olive oil, high in carbohydrates mainly from legumes and high in fibre) in healthy adults (Chrysohoou et al. 2004). A recent study with 125 older subjects, RISTOMED, has reported a similar anti-inflammaging effect of a mediterranean diet with or without supplementation with d-limonene (Ostan et al. 2016). Increased intake of specific dietary components, such as omega 3 fatty acids (Molfino et al. 2014) also has anti-inflammatory effects. Interestingly, the adoption of the mediterranean diet by older adults has also been associated improvement in immune responses, particularly dendritic cell function (Clements et al. 2017).

Another specific dietary component thought to possess immunomodulating properties are probiotics (Thomas and Versalovic 2010). Ageing is associated with a reduction in beneficial microbes in the colon including *Bifidobacteria,* countered by a rise in proteolytic bacteria (Pae et al. 2012). The administration of *B. bifidium* exerts an anti-senescence (reduced p16 expression in thymus and spleen) and anti-inflammatory effects (lower IL6 and TNFα levels) in old mice (Fu et al. 2010). A study done on 61 healthy adults aged > 65 years reported that probiotic consumption for 6 months increased the number of recent thymic emigrants and decreased the number of senescent CD8 CD28^null^ T cells (Moro-García et al. 2013). Additionally o an improved cytotoxicity of NK cells (Gill et al. 2001) and phagocytic activity of granulocytes (Maneerat et al. 2014) has been reported with probiotic consumption for 3 weeks in healthy elderly. Probiotic supplementation for 13 weeks has also been reported to improve vaccine responses in a clinical trial done in over 200 subjects > 70 years (Boge et al. 2009). Another trial in 1072 healthy old participants reported a reduced risk of respiratory infection in participants consuming fermented dairy product containing probiotics for 3 months (Guillemard et al. 2010).

Vitamin D has also emerged as a key modulator of a range of immune functions including: monocyte differentiation into macrophages, enhanced phagocytosis by macrophages, reduced production of pro-inflammatory cytokines by macrophages, suppression of DC maturation to promote tolerance, inhibition of Th1 and Th17 responses and regulation of B cell proliferation (reviewed in Hewison 2010; Vanherwegen et al. 2017). Vitamin D serum levels vary with the time of year but are also low in older adults (Gallagher 2013). An observational study reported a significant association between low vitamin D status and markers of inflammation in older adults (Laird et al. 2014). Although the potential of Vitamin D supplementation in reversing immunesenescence still remains largely unexplored, vitamin D supplementation has been reported to boost response to acute infections in age-associated inflammatory disorders (Yin and Agrawal 2014) and improve macrophage antibacterial properties in chronic kidney disease (Bacchetta et al. 2014).

Zinc is a trace element required for multiple immune cell tasks including suppression of production of pro-inflammatory cytokines (IL1β, TNFα) by monocytes/macrophages and decreasing reactive oxygen species (ROS) (Prasad 2009). A significant proportion of older adults have low serum zinc levels due to inadequate intake, impaired metabolism, infection and inflammation (Pae et al. 2012). Zinc deficiency has been reported to affect multiple immune cells and remarkably parallels changes in immune functioning with age including: impairments in neutrophil function (Haase and Rink 2009), macrophage phagocytosis (Rink and Gabriel 2000), NK cell cytotoxicity (Mocchegiani and Malavolta 2004), thymic involution (Mitchell et al. 2006), imbalance of Th1/Th2 differentiation (Prasad 2000), impairments in lymphocyte proliferation, IL2 production (Rink and Gabriel 2000) and decreased vaccine responses (Haase and Rink 2009). Zinc deficient subjects have also been shown to have greater susceptibility to pathogens (Walker and Black 2004). Importantly zinc supplementation has been shown to reduce infection incidence in older adults (Prasad et al. 2007) and has many effects indicative of reversal of immunesenescence including: improved NK cell cytotoxicity (Mocchegiani et al. 2003), modification of Th1/Th2 balance (Uciechowski et al. 2008), restoration of serum thymulin activity (Boukaïba et al. 1993) and improved vaccine responses (Duchateau et al. 1981).

## Pharmacological interventions to reduce immunesenescence

### Caloric restriction mimetics

Even though increasing lifespan and improving health in old age is enormously attractive to most people, a lifetime commitment to a reduced calorie diet is unlikely to be adopted at a population level. As a result, CR mimetics are being sought and several have already been identified including; Resveratrol (SIRT1 activator), Metformin (AMP kinase activator) and Rapamycin (mTOR inhibitor).

Resveratrol, a polyphenolic sirtuin activator (De la Lastra and Villegas 2005) which has been shown to extend lifespan in several species, occurs naturally in various plants including red grapes, peanuts and berries. Although there is not a vast literature on the impact of resveratrol on immunity in vivo, several in vitro studies have demonstrated immune modulating effects, these include a suppressive effect of resveratrol on neutrophil chemotaxis, superoxide generation (Cavallaro et al. 2003), T cell proliferation and cytokine production (Gao et al. 2001) and an enhancement of NK cell cytotoxicity (Lu and Chen 2010). Resveratrol’s immunomodulating properties are dose dependent manner, with low concentrations exerting a positive effect and higher concentrations being largely inhibitory (Falchetti et al. 2001). Additionally, anti-inflammatory effects of resveratrol have been reported in animal studies (Das and Das 2007; Tung et al. 2015). Another example of resveratrol as an anti-inflammatory agent has been highlighted in a study showing that the long-term treatment with resveratrol significantly attenuated the development of senescence-associated secretory phenotype (SASP) in senescent fibroblasts; reducing the release of proinflammatory cytokines (Pitozzi et al. 2013) by modulation of mRNA splicing (Latorre et al. 2017). Resveratrol is inexpensive and commercially available but studies in healthy humans are currently limited (Gualdoni et al. 2014).

Rapamycin (mTOR inhibitor), has emerged as another candidate that mimics the effects of CR. Reduced signalling through mTOR has been associated with increased longevity in invertebrates (Powers et al. 2006), as well as mammalian species (Harrison et al. 2009). Rapamycin is used clinically as an immune suppressant in transplant patients and mTOR mediated immunosuppression is mediated by modulation of effector and regulatory CD4 T cell subsets (Araki et al. 2011). To date there has been one placebo controlled trial in humans that has used low doses of a m-TOR inhibitor (RAD001) given daily for 6 weeks before the influenza vaccination in older adults. The treatment was shown to increase the antibody response by 20% for 2 out of 3 strains (Mannick et al. 2014) with few side effects. This drug may have benefits for immunity in old age as well as broader positive health effects through mTOR modulation.

Metformin, a treatment for type 2 diabetes, is able to mimic CR through its activation of AMP kinase and has also been shown to extend lifespan and healthspan in several species including rodents (Martin-Montalvo et al. 2013). Clinical data have also revealed that diabetics taking metformin have significantly lower mortality than diabetics on alternative sulphonyl urea therapies (Campbell et al. 2017). With regard to immunity, recent clinical studies have reported an anti-inflammatory role of metformin (Saisho 2015) and an attenuating effect of metformin on Th17 cell generation and upregulation of Tregs in mice models of arthritis (Son et al. 2014).

### Reversal of thymic atrophy

Thymic capacity is modulated by a range of positive (IL7, KGF, GH) and negative (TNF, IL6) factors (Ventevogel and Sempowski 2013). Thymic regeneration or maintenance would be a rational target for improving immune competence in older adults. Potential mechanisms for thymic regeneration include; IL7 or growth hormone replacement therapy (Aspinall and Mitchell 2008), enhanced keratinocyte growth factor signalling (Seggewiss et al. 2007). Studies carried out in old mice have reported that IL7 therapy reversed thymic atrophy, increased thymopoiesis and raised numbers of naïve T cells in blood (Aspinall et al. 2007; Henson et al. 2005). IL7 administration in preliminary human studies produced an increase in thymic T cell output and expansion of naïve T cells in patients with lymphopenia, cancer, chronic viral infections and following transplant (Mackall et al. 2011). IL7 has also been identified as an effective vaccine adjuvant that can augment antigen specific responses post vaccination recombinant lentivector immunised mice (Colombetti et al. 2009). The clinical evidence so far in humans has suggested that rhIL7 is safe with minimal side effects (Lundström et al. 2012), providing compelling evidence for testing rhIL7 as a therapeutic agent to restore thymic output in healthy older adults. Recently IL22 has been identified as another potential target for restoring thymic function due to its ability to promote proliferation and survival of thymic epithelial cells, supporting a microenvironment required for thymopoiesis (Chaudhry et al. 2016). Additionally, exogenous administration of recombinant GH has been reported to promote thymus regrowth in HIV infected adults (Napolitano et al. 2008), though the increased risk of cancer with GH may preclude its long term use.

### Statins (HMG-CoA reductase inhibitors)

The beneficial effect of statins in prevention of cardiovascular events by blocking cholesterol synthesis is well established (Thavendiranathan et al. 2006). Recent studies have shown that statins also possess anti-inflammatory properties and can reduce inflammatory markers, especially IL6 and CRP both during chronic inflammatory conditions (Nawawi et al. 2003; Montecucco and Mach 2009) and also in healthy older individuals (Mora et al. 2010) and this widely used drug may be a promising intervention for combating inflammaging. Our recent data has reported a beneficial of effect of statins in vitro and in vivo in restoring neutrophil migratory accuracy which declines with advancing age (Sapey et al. 2017). That this may be clinically relevant is supported by data showing that patients admitted to hospital with pneumonia who are already on statin medication have reduced mortality compared to those not taking statins (Grudzinska et al. 2017; Bruyere et al. 2014). Additionally, statins are now also recognised as modulators of telomerase activity in study conducted on 230 older adults and can slow telomere shortening, they showed that with every 1 year increment in age a decline by 0.058 Kb was observed in the no statin group compared with 0.033 Kb in the statin group (Boccardi et al. 2013). However, a major side effect of using statins that needs to be considered is statin-induced myopathy which is the most common cause of statin discontinuation and has been observed in 10–15% of statin users (Abd and Jacobson 2011).

### PI3kinase inhibitors

Neutrophil functions such as chemotaxis, phagocytosis and superoxide generation are regulated via phosphoinositide 3 kinase (PI3K) activity (Hannigan et al. 2004). Inaccurate neutrophil chemotaxis in old donors has been associated with increased constitutive PI3Kδ signalling and inhibitors of this pathway restore migrational accuracy with no negative effects on other neutrophil functions (Sapey et al. 2014). PI3K blocking therapies offer a new strategy to improve neutrophil function in older adults and might help improve outcomes during infection and reduce inflammation (Naccache and Lefebvre 2014). However, one potential drawback of using PI3K inhibitors is that they might have a negative effect on other immune cells, for instance PI3K is required for DC phagocytosis and migration. Aged DCs in contrast to neutrophils show decreased activation of the PI3K pathway with age (Agrawal et al. 2007) and a further suppression might be detrimental.

### p38 MAP kinase inhibition

T cells with senescent characteristics increase with age and exhibit constitutive p38 MAPK activation (Henson et al. 2015), with recent data revealing that this is due to formation of a complex between MAP kinases and the sestrin family of proteins which results in kinase activation (Lanna et al. 2017). Furthermore, knockdown of sestrins (Lanna et al. 2017) or inhibition of p38 MAP kinase (Lanna et al. 2014) restored T cell proliferative capacity and old sestrin knockout mice showed improved responses to vaccination compared with wild type mice (Lanna et al. 2017). Clinical trials using p38 inhibitors have been tested for a short time in inflammatory conditions such as rheumatoid arthritis, chronic obstructive pulmonary disease and there were no reports of increased risk of malignancy (Patterson et al. 2014), which is a concern in restoring proliferative capacity more generally. Interestingly TNFα has been shown to inhibit neutrophil migration via activation of the p38MAP kinase pathway (Lokuta and Huttenlocher 2005), suggesting that this pathway could also targeted to restore age associated defects in neutrophil function.

### IL15 therapy

IL15 is known to play a role in regulating immune homeostasis and acts as a lymphocyte survival factor, especially for naïve T cells (Wallace et al. 2006). IL15 also plays a critical role in the development and maintenance of NK cells (Cooper et al. 2002) and as stated above NK cell cytotoxicity declines with age (Hazeldine et al. 2012). In vitro studies examining the effect of IL15 on NK cells from acute myeloid leukaemia patients reported an increase in expression of NK cell receptors NKp46 and NKp30 (Sanchez-Correa et al. 2016). Clinical evaluation of IL15 in cancer therapy has shown to induce expansion of local T cells and infiltration of long-lived memory T cell capacity (Pilipow et al. 2015). These findings suggest the possibility of using cytokine modulation to improve NK cell responses and increase naïve: memory T cell ratio in older adults.

## Conclusion

**Fig. 3:**
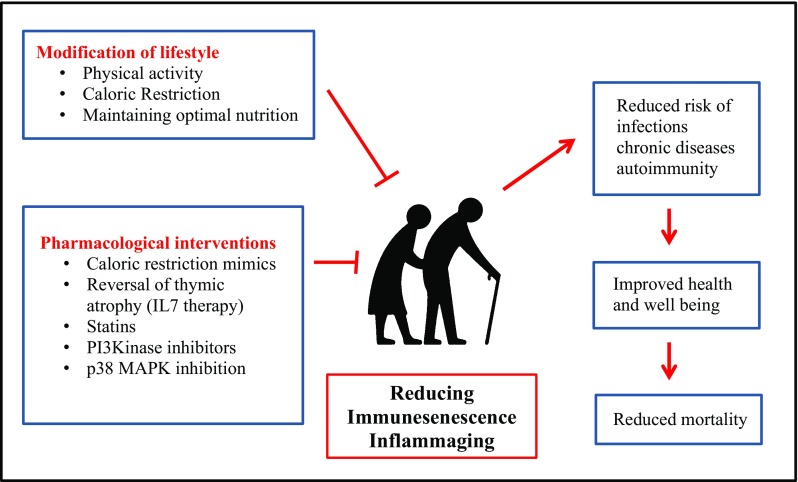
Intervention strategies targeting immunesenescence and inflammaging

The immune system is substantially remodeled with ageing leading to a decline in efficacy with advancing age, resulting in increased risk of chronic diseases, infections, autoimmunity and vaccine failure. Changes in nutrition and lifestyle can be an effective approach towards improving immune outcome in older adults but may be hard to achieve at a population level (Fig. 3). Research in the field of interventions to target immune senescence is gathering pace and improving immune responses such as vaccinations may be used as an early biomarker for anti-ageing effects. A wide range of pharmacological agents with anti-immunesenescence properties have been identified (Fig. 3) and trials with agents such as rapamycin analogs are underway. Thus, immunomodulation represents a promising therapeutic approach to improve the health of older adults.
